# Artificial Intelligence Versus Human Dental Expertise in Diagnosing Periapical Pathosis on Periapical Radiographs: A Multicenter Study

**DOI:** 10.3390/bioengineering13020232

**Published:** 2026-02-17

**Authors:** Fatma E. A. Hassanein, Radwa R. Hussein, Mohamed Riad Elgarhy, Shaymaa Mohamed Maher, Ahmed Hassen, Sherif Heidar, Marwa Ezz El Arab, Amr Edress, Asmaa Abou-Bakr, Mohamed Mekhemar

**Affiliations:** 1 Oral Medicine, Periodontology, and Oral Diagnosis, Faculty of Dentistry, King Salman International University, El Tur 46612, Egypt; 2 Oral Medicine and Periodontology, Ain Shams University, Cairo 11566, Egypt; 3 Endodontics Department, Faculty of Dentistry, Galala University, Suez 43511, Egypt; 4 Faculty of Dentistry, King Salman International University, El Tur 46612, Egypt; 5 Endodontic Department, Faculty of Dentistry, Suez University, Suez 43511, Egypt; 6 Endodontic Department, Faculty of Dentistry, King Salman International University, El Tur 46612, Egypt; 7 Oral Radiology, Faculty of Dentistry, British University in Egypt, Cairo 11837, Egypt; 8 Conservative Dentistry Department, Faculty of Dentistry, King Salman International University, El Tur 46612, Egypt; 9 Oral Medicine and Periodontology, Faculty of Dentistry, Galala University, Suez 43511, Egypt; 10 Clinic for Conservative Dentistry and Periodontology, School of Dental Medicine, Christian-Albrecht’s University, 24118 Kiel, Germany

**Keywords:** artificial intelligence, periapical radiography, periapical periodontitis, diagnosis, deep learning, diagnostic accuracy, ChatGPT

## Abstract

**Background**: Periapical pathosis in periapical radiographs must be properly diagnosed for the success of endodontic treatment but is often muddled by 2D imaging limitations and subjective interpretation. Artificial intelligence (AI) offers a solution, but whether the diagnostic granularity of AI versus human clinicians in everyday clinical practice has been adequately explored remains to be addressed. The purpose of this study was to evaluate the diagnostic accuracy of ChatGPT-5 in detecting periapical radiographic abnormalities compared with the three-expert consensus reference standard. **Methods**: In this diagnostic accuracy retrospective study, 270 periapical radiographs were independently read by a large language model (ChatGPT-5) and a three-board-certified oral radiologist consensus. The AI was given a standardized prompt to label radiographic features, like the presence of periapical radiolucency, border, shape, and integrity of lamina dura. Diagnostic accuracy, agreement (Cohen’s κ), and predictors of correct AI classification were compared with the expert consensus reference standard. **Results**: ChatGPT-5 demonstrated high sensitivity (87.5%) but low specificity (12.5%), resulting in an overall diagnostic accuracy of 50.0%. This performance profile reflects a tendency toward over-identification of pathology, with the model classifying 87.5% of radiographs as abnormal compared with 50.0% by expert consensus. Agreement was almost perfect for anatomical localization (arch, κ = 0.857) but poor for binary abnormality detection (κ = 0.000). For morphological descriptors, statistically significant disagreement was observed for lesion border characterization (κ = 0.127; *p* < 0.001), whereas lesion shape demonstrated only descriptive divergence without reaching statistical significance (κ = 0.359). Root resorption assessment also differed significantly between evaluators (*p* = 0.046). Regression analysis showed that well-defined corticated borders (OR = 60.25, *p* < 0.001) and first molar-associated lesions (OR = 32.55, *p* < 0.001) were significant predictors of correct AI classification. **Conclusions**: This study demonstrates that while ChatGPT-5 Vision can visually interpret periapical radiographs with high sensitivity, limited specificity and inconsistent morphological feature characterization restrict its reliability for independent clinical diagnosis. The AI system tends to over-diagnose systematically and categorizes lesions more structurally and defined compared to dental experts. AI has the potential for being optimized as a sensitive first-screening test, but its findings must be validated by dental professionals to avoid false positives and ensure proper characterization.

## 1. Introduction

Periapical pathosis (periapical granulomas, radicular cysts, abscesses) is a frequent sequel to endodontic infections that affect approximately 5–52% of the world’s adult population [1]. Correct differential diagnosis of these lesions is important for adequate treatment, which is key to long-term success in endodontic treatment. However, clinical diagnosis is challenging due to the subjective nature of radiographic interpretation and its inherent imaging limitations [2,3,4,5].

In general practice, the most available and used imaging modality is that of the two-dimensional (2D) periapical radiograph, which has inherent limitations, such as the anatomical structures that overlap the periapical regions may cover or simulate such lesions, resulting in loss of diagnostic clarity [6]. Additionally, the diagnostic impressions frequently differ between clinicians, and the literature reports substantial inter-observer variability [7]. These issues are further compounded by cases being referred too late or incorrectly, as studies indicate that cone-beam computed tomography (CBCT) can detect more lesions but is underutilized because of accessibility, radiation dose, and cost barriers [8].

With the recent advances in artificial intelligence (AI), there are high expectations that AI will play a significant role in dental diagnostics. The sensitivity of deep learning models in detecting periapical lesions on radiographs has been reported as 92% to 98% [9,10]. However, several of these methods focus on binary classification (i.e., healthy vs. disease) and do not provide sufficient diagnostic granularity to discriminate between different pathoses, such as granulomas, cysts, or abscesses [11,12]. This limitation significantly reduces their clinical usefulness, as treatment planning relies on precisely these morphologic differences.

The recent emergence of general-purpose, multimodal large language models (LLMs) like ChatGPT is a paradigm break with task-based, specialized AI [13,14,15,16,17,18]. These models have generalized reasoning capabilities but pose a huge, untested issue: can a general-purpose AI system, not specifically trained on dental radiographs, match the nuanced diagnostic intuition of a specialist? While there are a few reported studies on validating the AI performance, particularly the LLMs, versus the assessments of experienced endodontic specialists in the clinical routine setting [19,20,21,22,23], a significant gap remains between strong, clinically-grounded evidence of these models and a multi-expert consensus for the fine-grained, feature-by-feature comparison that underlies radiographic diagnosis. Most literature has focused on differential diagnosis generation in difficult cases rather than considering the model’s fundamental competence in identifying and describing specific radiographic features, such as lesion borders, shape, and lamina dura integrity, within the typical clinical setting.

Therefore, this multi-center study was designed to bridge this significant gap by conducting a feature-level thorough evaluation of ChatGPT-5. We aimed to move beyond crude binary detection and compare the model’s performance to a three-expert consensus reference standard in classifying the most significant morphological descriptors of periapical pathosis on 2D radiographs. To improve workflow efficiency, reduce diagnostic variability, and facilitate informed decision-making in the diagnosis of periapical pathoses, this study aimed to evaluate the clinical utility of ChatGPT-5. Accordingly, this investigation was designed as a stress test and proof-of-concept evaluation of a general-purpose multimodal LLM under non-specialized deployment conditions, rather than as a direct performance comparison with task-optimized CNN-based diagnostic systems.

## 2. Materials and Methods

### 2.1. Study Design and Reporting Framework

This investigation was a retrospective diagnostic accuracy study, designed and reported according to the STARD 2015 guidelines for diagnostic accuracy research [24]. The study compared the performance of a large language model ChatGPT-5 (Released in 2025) in detecting radiographic features of periapical pathology against an expert consensus used as the reference standard.

### 2.2. Study Setting and Data Sources

Radiographic data were obtained from the digital imaging archives of four private dental clinics, comprising anonymized periapical radiographs acquired between January 2024 and August 2025. All images were captured using standardized intraoral radiographic techniques, following consistent exposure parameters and image-quality assurance protocols applied at the clinic. The study was conducted as a retrospective diagnostic accuracy analysis using pre-existing radiographs. Ethical approval was reviewed and an exemption granted by the Research Ethics Committee of the Faculty of Dentistry, Ain Shams University Exemption No. (FDA SU-REC: IR072509), in accordance with the Declaration of Helsinki and institutional guidelines for secondary analysis of fully anonymized, non-identifiable patient data. Because all data were completely anonymized and no patient identifiers were accessible to the investigators, individual informed consent was not required.

### 2.3. Sample Size Calculation

The study was powered to estimate the sensitivity and specificity of the index test (ChatGPT-5) against expert consensus with sufficient precision, rather than relying solely on overall accuracy, which is influenced by disease prevalence. Sample size was determined using Buderer’s precision-based method for diagnostic accuracy studies, which incorporates expected prevalence, sensitivity, and specificity [25]. Assuming an expected sensitivity of 0.85, specificity of 0.80, and a desired precision of ±0.07, the minimum required sample sizes were 100 abnormal radiographs and 126 normal radiographs. Allowing approximately 10% for potential exclusions and subgroup analyses, the final planned sample comprised about 276 radiographs. This target was consistent with prior AI diagnostic accuracy studies using periapical radiographs [26].

### 2.4. Participants and Eligibility Criteria

#### 2.4.1. Inclusion Criteria

**Figure 1:**
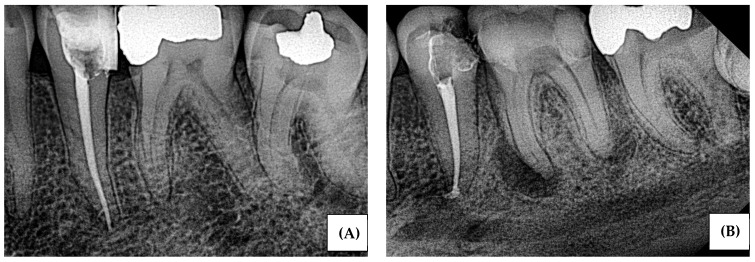
Example periapical radiographs: (**A**) abnormal image with periapical radiolucency; (**B**) normal image with intact lamina dura and no periapical radiolucency.

Adult patients (≥18 years) with periapical radiographs demonstrating full visualization of the tooth apex and surrounding periapical bone. Eligible images included radiographs with either (1) normal periapical appearance characterized by an intact lamina dura and uniform periodontal ligament space, or (2) periapical radiolucency consistent with periapical pathosis. All images were acquired using standardized intraoral periapical techniques, met institutional quality-control standards, were fully anonymized, and were eligible for secondary analysis. Representative normal and abnormal examples are provided in Figure 1.

#### 2.4.2. Exclusion Criteria

Radiographs were excluded if obtained from patients with systemic conditions or medications known to alter bone density or periapical bone architecture (e.g., osteoporosis, metabolic bone disease, long-term corticosteroid therapy, or uncontrolled diabetes mellitus). Images exhibiting severe artifacts (motion, metallic, prosthetic, or processing-related artifacts), incomplete visualization of the root apex, image cropping, or superimposition obscuring the periapical region were also excluded. Additionally, overexposed, underexposed, or low-contrast radiographs that compromised diagnostic interpretability, as well as duplicated or follow-up images of the same tooth or patient, were excluded.

### 2.5. Index Test (ChatGPT Assessment)

The index test consisted of radiographic feature interpretation performed by ChatGPT-5 Vision (OpenAI, released August 2025). The model was accessed through the standard ChatGPT platform in a controlled research setting. As the model is cloud-based and continuously updated, it does not have a static build number. The platform operates under system-controlled inference settings; therefore, no manual adjustment of temperature, sampling parameters (top-p), or token limits was possible, and the model functioned under its default deterministic configuration. Each periapical radiograph was directly uploaded into the ChatGPT-5 Vision interface and analyzed as a visual input using the model’s multimodal vision-language architecture. The model independently interpreted the radiographic image and generated structured diagnostic outputs based on its own visual assessment. No pre-existing textual reports, annotations, or expert descriptions were provided to the system.

### 2.6. Prompt Used for ChatGPT-5 Assessment

“You are an experienced oral and maxillofacial radiologist. Examine the following periapical radiograph and identify any radiographic abnormalities. Specifically, report the presence or absence of

1. Periapical radiolucency (normal vs. abnormal)
2. Arch (upper or lower)
3. Position (anterior or posterior)
4. Tooth involved
5. Lesion borders (ill-defined, well-defined corticated, well-defined non-corticated)
6. Lesion shape (irregular, round, or oval)
7. Lamina dura integrity (lost or intact)
8. Root resorption (present or absent)
9. Cortical expansion (present or absent)
10. Tooth displacement (present or absent)

Provide your output as a structured list using the same categories, without additional commentary or interpretation.”

The instruction explicitly required direct visual examination of the uploaded radiographic image and did not involve any intermediate textual description of radiographic findings. ChatGPT output was recorded verbatim and exported for analysis without modification. No additional clarifying prompts or iterative refinements were allowed in order to maintain diagnostic consistency and blinding integrity. ChatGPT-5 had no access to patient metadata, prior annotations, or expert reference results, ensuring full independence of interpretation.

### 2.7. Reference Standard (Expert Consensus)

The reference standard was established through the consensus of three board-certified oral and maxillofacial radiologists, each with over 10 years of clinical and academic experience in diagnostic dental imaging. All experts independently evaluated the same set of periapical radiographs under identical viewing conditions using the same diagnostic framework applied to the AI model. Each radiograph was assessed for: periapical radiolucency (normal vs. abnormal), arch (upper or lower), position (anterior or posterior), tooth involved, lesion borders, shape, lamina dura integrity, root resorption, cortical expansion, and tooth displacement.

All experts were blinded to ChatGPT-5 outputs and to each other’s evaluations. Image presentation order was randomized before assessment to minimize potential interpretive bias. Following an independent review, discrepant cases were resolved by consensus discussion involving all three radiologists. In instances where unanimous agreement could not be reached, the final diagnosis was determined by majority vote (2 out of 3 raters), consistent with established practices in diagnostic accuracy research. Because the reference standard was consensus-based, formal inter- or intra-rater reliability testing was not applicable.

### 2.8. Outcome Assessment

The primary outcome of this study was the diagnostic accuracy of ChatGPT-5 in detecting periapical radiographic abnormalities compared with the three-expert consensus reference standard. Diagnostic accuracy was evaluated at both the binary level (normal vs. abnormal radiograph) and the feature level, covering lesion borders, shape, lamina dura integrity, root resorption, cortical expansion, and tooth displacement.

Each ChatGPT-5 output was matched against the corresponding expert consensus result. Performance metrics were derived from the confusion matrix, including the following:

- Sensitivity (true-positive rate)—proportion of abnormal radiographs correctly identified by ChatGPT-5.
- Specificity (true-negative rate)—proportion of normal radiographs correctly identified.
- Positive Predictive Value (precision)—proportion of AI-identified abnormalities that were true positives.
- F1 score—harmonic mean of sensitivity and precision.
- Balanced accuracy—average of sensitivity and specificity to correct for class imbalance.
- Overall accuracy—proportion of correct classifications.

Secondary outcomes included agreement analysis using Cohen’s κ and prevalence- and bias-adjusted κ (PABAK) for each categorical radiographic parameter.

For exploratory analysis, binary logistic regression identified radiographic and anatomical predictors influencing ChatGPT-5’s correct detection relative to the expert consensus. Odds ratios (ORs) with 95% confidence intervals (CIs) were computed using the Haldane–Anscombe correction for sparse cells.

### 2.9. Statistical Analysis

All analyses were performed using R (version 4.3.2; R Foundation for Statistical Computing, Vienna, Austria) and IBM SPSS Statistics for Windows (version 23.0; IBM Corp., Armonk, NY, USA). Descriptive data were summarized as counts and percentages. Paired categorical comparisons between ChatGPT and expert assessments were conducted using McNemar’s test for binary outcomes and Bowker’s test of symmetry for multicategory variables. Diagnostic performance indices (sensitivity, specificity, precision, F1 score, and balanced accuracy) were calculated with 95% confidence intervals. Agreement between raters was assessed using Cohen’s κ, supplemented by prevalence- and bias-adjusted κ (PABAK) where appropriate. Statistical significance was set at *p* < 0.05 for all analyses.

## 3. Results

### 3.1. Study Sample and Image Characteristics

**Figure 2:**
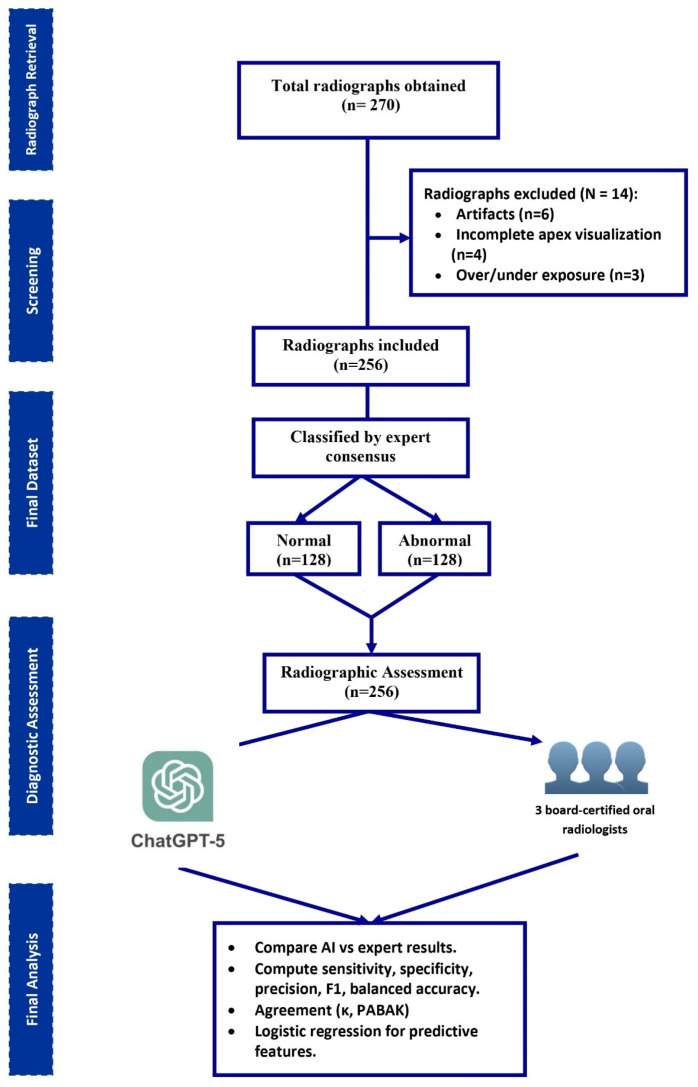
Study design.

A total of 270 periapical radiographs were initially screened. After excluding 14 images with incomplete visualization or overlapping anatomy, 256 radiographs remained for analysis (Figure 2). The images were sourced from four independent private dental clinics, each contributing 64 anonymized periapical radiographs. Expert consensus classified 128 radiographs (50.0%) as abnormal and 128 (50.0%) as normal. ChatGPT-5 identified 224 (87.5%) as abnormal and 32 (12.5%) as normal, indicating a tendency toward over-identification of pathology.

### 3.2. Descriptive Distribution of Radiographic Features

Experts classified approximately half of the evaluated radiographs as abnormal, whereas ChatGPT-5 labeled a substantially higher proportion, indicating systematic over-diagnosis. Both raters showed predominance of posterior involvement, with first molars most frequently affected. Compared with experts, ChatGPT-5 more frequently assigned well-defined, corticated borders and over-identified cortical expansion and tooth displacement, while experts reported higher rates of lamina-dura loss and root resorption. These patterns indicate a model bias toward structured lesion morphology and abnormal classification (Table 1).

### 3.3. Diagnostic Accuracy Metrics

Using expert consensus as the reference standard, ChatGPT correctly identified 112 abnormal and 16 normal radiographs, yielding an overall accuracy of 50.0%. The model demonstrated high sensitivity (87.5%), indicating effective detection of abnormal lesions, but markedly low specificity (12.5%), reflecting over-classification of normal images as abnormal. Precision (PPV) reached 50.0%, consistent with a balanced proportion of true- and false-positive predictions, while the F1 score (63.6%) highlighted moderate harmonic performance between sensitivity and precision. The balanced accuracy (50.0%) confirmed a neutral performance profile when accounting for both sensitivity and specificity.

**Figure 3:**
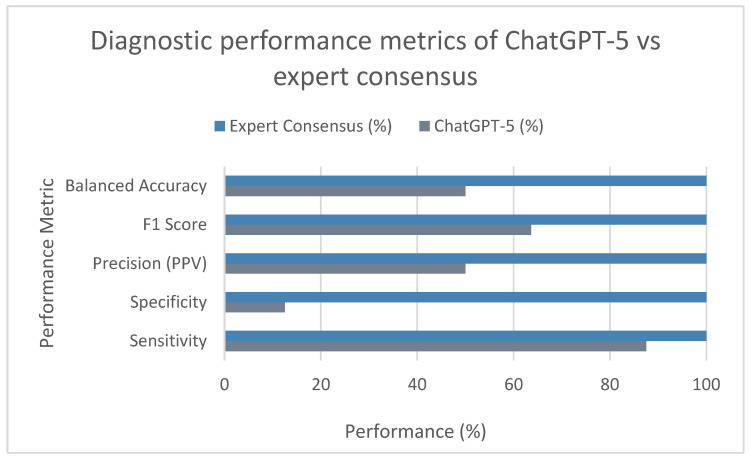
Diagnostic performance metrics of ChatGPT vs. expert consensus (bar plot). PPV = positive predictive value; F1 = harmonic mean of precision and recall; BA = balanced accuracy.

**Figure 4:**
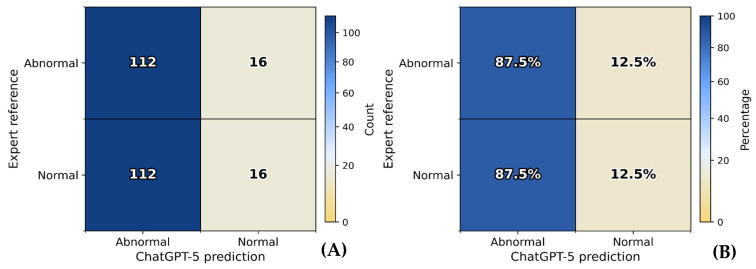
Confusion matrix of ChatGPT-5 versus expert reference standard. TP = true positives, FP = false positives, TN = true negatives, FN = false negatives. (**A**) Absolute counts of normal and abnormal classifications. (**B**) Row-normalized percentages showing 87.5% sensitivity and 12.5% specificity, indicating ChatGPT-5’s tendency to over-classify abnormalities.

Collectively, these results indicate that ChatGPT favored a sensitivity-oriented diagnostic strategy successfully detecting most abnormal cases but at the expense of substantial false positives, mirroring an overdiagnosis bias toward abnormality recognition (Table 2; Figure 3 and Figure 4).

### 3.4. Agreement Between ChatGPT-5 and Expert Consensus

Agreement varied substantially across radiographic parameters (Table 3). Overall abnormality detection showed poor agreement (κ = 0.000), reflecting systematic disagreement despite equal prevalence. Anatomical localization demonstrated strong consistency, with almost perfect agreement for arch (κ = 0.857) and moderate agreement for tooth position and tooth-specific localization. In contrast, morphologic descriptors showed limited reliability, with slight agreement for lesion borders and root resorption and fair agreement for lesion shape. Lamina-dura status and tooth displacement exhibited high raw agreement but near-zero or negative κ values, indicating prevalence-related instability rather than true concordance. Overall, ChatGPT-5 aligned with experts in anatomical localization but diverged markedly in morphologic interpretation, particularly for lesion margins and structural changes.

### 3.5. Comparative Tests of Radiographic Parameters

Among radiographs classified as abnormal by expert consensus (n = 128), ChatGPT-5 demonstrated selective disagreement across radiographic descriptors (Table 4). Significant differences were observed for lesion border characterization (*p* < 0.001) and root resorption detection (*p* = 0.046), whereas arch location, tooth position, tooth type, lesion shape, lamina-dura status, cortical expansion, and tooth displacement did not differ significantly between raters (all *p* > 0.05). ChatGPT-5 more frequently assigned well-defined, corticated margins and classified resorption as present, while experts reported higher frequencies of ill-defined lesion borders. Overall, these findings indicate that disagreement was primarily driven by morphologic feature interpretation rather than anatomical localization.

### 3.6. Predictors of Correct Classification (Regression Analysis)

Exact binary logistic regression identified multiple radiographic and anatomical predictors significantly associated with ChatGPT’s diagnostic accuracy relative to expert consensus. Lesions with well-defined corticated borders showed the strongest association with correct classification (OR = 60.25, 95% CI 3.62–1002.64, *p* < 0.001), followed by oval-shaped lesions (OR = 20.43, *p* = 0.003) and well-defined non-corticated borders (OR = 3.46, *p* = 0.004). An intact lamina dura significantly reduced the odds of correct detection (OR = 0.03, *p* < 0.001), indicating ChatGPT’s tendency to under-detect subtle pathology. Cortical expansion improved the accuracy slightly (OR = 2.10, *p* = 0.046), whereas root resorption and tooth displacement were non-significant. Regarding anatomical factors, a trend was observed toward better performance in the lower arch (OR = 1.61, *p* = 0.092), while position showed no effect. Importantly, tooth involvement was a major predictor: lesions around first molars were over 30 times more likely to be correctly classified (OR = 32.55, *p* < 0.001), reflecting clearer radiographic landmarks and fewer superimpositions in posterior regions (Table 5).

Collectively, the regression model demonstrates that distinct morphologic features and tooth-region context, particularly corticated borders, oval contours, and molar localization, substantially enhance ChatGPT’s diagnostic agreement with expert radiographic interpretation.

ChatGPT-5 demonstrated high sensitivity but low specificity, producing frequent false-positive classifications. Agreement with expert consensus varied by feature type—strong for anatomical localization, moderate for lesion shape, and weak for morphologic detail. The model’s accuracy improved substantially for lesions with distinct, corticated borders and oval shapes, indicating a pattern-recognition bias toward regular and well-demarcated structures.

## 4. Discussion

This study provides the first feature-level diagnostic accuracy evaluation of a general-purpose multimodal language model applied to periapical radiographic interpretation. The findings highlight the performance limitations of ChatGPT-5 in the evaluated general-purpose configuration when applied to subtle radiographic diagnostic tasks without specialized domain adaptation. Our results indicate that, in its current general-purpose configuration and without domain-specific radiographic training, ChatGPT-5 demonstrated limited suitability for independent diagnostic use in periapical radiographic interpretation. This lack of suitability manifests itself in a routine over-diagnosis of pathology and continual failure to recognize the critical morphological characteristics on which endodontic clinical decision-making is based.

The most notable finding of the current research was the unambiguous imbalance in the diagnostic performance of ChatGPT-5, which established high sensitivity (87.5%) at the cost of clinically unfavorable low specificity (12.5%) and a marked over-diagnosis tendency, which is, in its very nature, affected by the nature of the model itself. Although ChatGPT-5 incorporates multimodal vision–language capabilities, its core architecture and optimization objectives differ from task-specific convolutional neural networks trained exclusively on medical imaging datasets. Thus, our study did not evaluate a dedicated pixel-level lesion detection network, but rather assessed ChatGPT-5’s multimodal vision–language reasoning pipeline, in which visual radiographic input is processed and translated into structured textual diagnostic outputs. This crucial distinction explains the model’s eagerness to mark descriptions as abnormal, which contributes to high false-positivity, as it lacks visual context to exclude clinically irrelevant variations.

Comparative studies on LLMs in radiology board exams have also found that although the models may demonstrate vast knowledge, they are poor at tasks involving higher-order thinking, i.e., applying specific concepts and calculations, and tend to give wrong answers with certainty [28]. This pattern extends to dental education, where a trained ChatGPT-4 was more accurate in text-based endodontic diagnoses than students [29], indicating its potential as an educational tool, but in a controlled, non-image environment. The root issue, as debated in LLM reviews of radiology, is that generalist models like ChatGPT are not learned on a specialty-specific database and therefore are prone to having “hallucinations” and oversimplifying complex, subtle clinical features [18,30,31]. Consequently, while we, together with others, confirm LLMs’ amazing capacities, together they all underscore their current worth in clinical radiology and challenging diagnostic practice as an ancillary device that has to be rigorously validated by human operators, rather than as a stand-alone decision-maker.

The performance pattern observed in our investigation—high sensitivity but limited specificity and fine-grained feature analysis—is duplicated in recent evaluations of general-purpose LLMs across a variety of medical imaging subspecialties. Similar performance patterns have been reported in recent evaluations of other multimodal large language models. Studies assessing GPT-4-based vision models in chest radiography and cross-sectional imaging have demonstrated moderate overall diagnostic accuracy with substantial variability across pathology types, particularly for subtle or low-contrast findings. These observations support the notion that current general-purpose LLMs, while capable of generating coherent image-based descriptions, remain limited in fine-grained radiographic feature discrimination when compared with domain-trained imaging models or expert clinicians.

For instance, Lacaita et al. (2025) assessed the interpretation of chest and abdominal X-rays by ChatGPT-4o, reporting moderate overall diagnostic accuracy of 69%, with performance varying considerably by pathology [32]. Similarly, Arruzza et al. (2024) evaluated the performance of ChatGPT-4o in critiquing radiographic positioning with limited accuracy, since only 20% of projections were critiqued completely correctly [33]. In combination with our findings, these studies collectively demonstrate that, while multimodal LLMs possess a remarkable potential to engage with medical images, their diagnostic reasoning is less accurate and consistent compared to domain-specific AI or human experts. The overall evidence underscores one basic limitation: generalist LLMs, without specialist radiological training, are likely to be very good at identifying overt, textbook abnormalities but less skilled at the subtle, nuanced, and often irregular features that form the basis of accurate clinical diagnosis and differential assessment in dentistry and general radiology.

Our findings, though, do reveal a significant distinction between specialized deep learning models and general-purpose LLMs. A prior work established that specialized AI can actually excel at diagnostic tasks for periapical disease.

For instance, Ekert et al. (2019) [26], demonstrated that a specialized deep learning model might possess high sensitivity in detecting apical lesions on panoramic radiographs. Additionally, Endres et al. (2020) [34] also presented high diagnostic performance of a deep learning algorithm for periapical disease detection, and a newer systematic review by Pul et al. (2024) [9] combined evidence showing that purpose-built AI models are capable of accurate identification of periapical radiolucency.

By contrast, within the context of this stress-test evaluation framework, our assessment of ChatGPT-5 reveals a different profile of performance—high sensitivity but very low specificity—that is qualitatively different. This indicates that the skills of domain-specific, specialized AI cannot be extrapolated to multimodal LLMs without special training and validation.

However, our tested specificity of 12.5% is a dramatic departure from the better-balanced performance values normally given in research into specialized AI models. The departure is indicative of a significant issue brought out by Krois et al. (2021) [35] regarding the “generalizability gap,” whereby AI models will not perform when exposed to data that is different from the training set.

Using a general-purpose multimodal LLM model in the current investigation, not exclusively trained on dental radiographs, is a reality check that confirms that, without domain-specific calibration, a generalist AI can be severely impacted by an over-diagnosis bias, a concern less frequently encountered in purpose-built models, medically tuned AI systems. In the study by Allihaibi et al. (2025) [36], for example, a commercial AI platform with a specific purpose showed high diagnostic accuracy for assessing endodontic outcomes on periapical radiographs; this profile was much more balanced than the generalist ChatGPT models.

Our findings contribute directly to novel literature evaluating generalist LLMs in imaging diagnosis. Initial tests with models like GPT-4V have reported promising but rather variable performance at interpreting medical images such as chest X-rays and CTs, generally highlighting their ability to generate coherent reports but describing limitations in precise, quantitative feature extraction [22,23]. Likewise, in dentistry, initial forays into LLMs have centered on how they can assist in differential diagnosis from text-based descriptions of cases, rather than on their core competency of granular radiographic feature analysis [20].

Our feature-level diagnostic accuracy evaluation shows that this broad generalist thinking ability has not yet been instantiated as a consistent, subtle radiographic interpretation. Statistically significant disagreement was limited to lesion border characterization, whereas lesion shape differences remained descriptive and did not reach statistical significance. Accordingly, morphological bias in ChatGPT-5 performance appears to be driven predominantly by margin interpretation rather than global lesion geometry. Granular feature-specific agreement shows more of the diagnostic reasoning of the AI. Anatomical localization (e.g., arch, κ = 0.857) is a virtual replica of the model’s competence in rudimentary structural identification, an exercise wherein AI has traditionally fared well [37]. Performance deterioration was most pronounced for lesion border characterization, which demonstrated statistically significant disagreement, whereas other morphological descriptors exhibited variable but predominantly non-significant descriptive trends.

Human experts, however, account for the often subtle and irregular character of inflammatory periapical pathosis, a subtlety integral to differential diagnosis. This is in line with Issa et al. (2023) [38], whose assessment noted that while promising, AI detection remains a challenge for in-depth characterization.

The preference of the model for crisp, textbook morphological forms is likely a reflection of its training on vast corpora of general text and images, where such unambiguous, canonical forms are plentiful. Conversely, its inability to uniformly recognize the more indistinct, ill-defined margins characteristic of so many inflammatory periapical lesions is simply a function of its lack of focused training on the subtle range of dental pathology.

This is not the first instance where this limitation in capturing subtle features has been discovered in another scenario. Ren et al. (2024) determined in their study of osteosarcoma diagnosis from X-rays that whereas ChatGPT-4 was quite good at identifying the presence or absence of a space-occupying lesion (Accuracy: 82.5%), its performance at generating a precise, specific diagnosis of osteosarcoma was weak (Accuracy: 67.5% for “priority” diagnosis), with a very low sensitivity of 35.0% [39]. This concurs with our result that the evaluated general-purpose configuration of ChatGPT-5 demonstrated difficulty with fine-grained discrimination. The same was also discovered by Suárez et al. (2025) who evaluated ChatGPT-4o’s performance in the analysis of orthopantomograms (OPGs) for the assessment of third molar and achieved a total accuracy of only 38.44% [40]. Their article stated that the model tended to provide “incomplete or made-up information,” especially in compound situations where the structures overlap, as our own research which found its over-simplified and otherwise inaccurate morphological accounts.

Our regression model identified well-corticated, defined borders (OR = 60.25) and oval shapes (OR = 20.43) as the most predictive of the AI’s diagnostic concordance with the experts. This shows that the model’s accuracy is highly context-dependent and based on clear, classic radiographic features being available. This finding is novel in the general-purpose LLM literature and shows their diagnostic reasoning to be based on salient, well-corticated features rather than a general set of subtle radiographic features.

The most compelling comparison is with Stephan et al.’s (2024) study, which looked at another related but different application: using ChatGPT to automatically generate radiology reports from pre-completed checkbox questionnaires [41]. Although report writing was their concern and not actual image reading, they had an equivalent core challenge: significant loss of diagnostic data. Their text-processed reports, while highly readable and perfect, omitted a great number of findings from the initial checklist. This is a fundamental limitation for LLMs in a clinical setting: whether reading images or generating text, they tend to oversimplify complex information, with a resulting loss of diagnostic nuance. Moreover, Mago and Sharma (2023) found that while ChatGPT-3 performed well in defining pathologies and anatomical landmarks based on text-based inputs, it was less detailed and had trouble with abbreviations, indicating a lack of deep, contextual medical exposure [42].

The challenges of anatomical noise and superimposition of 2D imaging appear to be addressed poorly by ChatGPT, leading to its grossly elevated false-positive rate. This substantiates the argument of Schwendicke, Samek, and Krois (2020) [43] that the success of AI hinges on how it is developed, trained, and to what end. Our experience with a generalist model stands in stark contrast to the successful functioning of specialist systems, and this serves to indicate that a “one-size-fits-all” ChatGPT is not yet suitable for high-stakes diagnostic use in dentistry.

A strength of this study is the prospective, multicenter design with a pre-determined sample size, which enhances the generalizability and statistical power of our findings. Also, in that expert consensus was utilized as the reference standard, rather than a single rater, which mitigates the impact of individual observer bias.

### 4.1. Limitations

There are a number of limitations that must be mentioned, however. First, the reference standard itself was obtained from 2D radiographs, which have inherent limitations. While expert consensus represents a clinically relevant comparator, the absence of a definitive gold standard such as histopathology or CBCT confirmation for all cases means that some true pathology may have been misclassified. Second, this study evaluated a single general-purpose multimodal LLM (ChatGPT-5). Its performance may not be directly comparable to specialized, medical-grade AI systems trained on curated dental radiographic datasets. Third, the continuously updated, cloud-based nature of ChatGPT-5 represents an important reproducibility limitation. Because model updates and inference optimizations are controlled by the provider and not version-locked, exact replication of the present experimental conditions may not be achievable over time, even by the original investigators. Consequently, the reported performance reflects the model state at the time of evaluation and should be interpreted accordingly. Future studies should report model access timestamps and archive prompt protocols to improve temporal traceability and partial reproducibility of multimodal LLM-based diagnostic evaluations. Finally, the “black box” nature of the model limits interpretability and restricts insight into the underlying mechanisms driving observed classification biases and errors.

### 4.2. Practical and Clinical Implications

The findings suggest that general-purpose AI such as ChatGPT-5 should be integrated into dental practice cautiously and strategically rather than as a stand-alone diagnostic tool. Its high sensitivity (87.5%) supports a potential role as a triage or pre-screening assistant, capable of flagging radiographs with possible abnormalities for prioritized human review, while images classified as “normal” may be processed more efficiently given the low false-negative risk. However, the extremely low specificity (12.5%) and marked over-diagnosis tendency introduce substantial false-positive burden, which could lead to unnecessary patient anxiety, additional confirmatory imaging with increased radiation exposure, and avoidable economic costs if AI outputs are acted upon without verification. Importantly, AI-generated “abnormal” findings must be framed strictly as preliminary screening signals rather than definitive diagnoses. Moreover, significant disagreement in morphological feature assessment—particularly lesion border characterization (κ = 0.127, p < 0.001)—underscores the model’s current limitation in differential diagnosis and treatment planning, areas that remain dependent on expert clinical judgment. Nonetheless, AI-assisted reporting may indirectly promote more standardized radiographic documentation through structured descriptive terminology. Overall, the appropriate path forward is one of complementarity: ChatGPT-5 may function as a sensitive alert system within a governed workflow, but definitive interpretation, diagnostic reasoning, and clinical decision-making must remain under professional supervision

### 4.3. Future Directions

Future research needs to be guided in several directions. First, fine-tuning general-purpose LLMs on large, annotated datasets of dental radiographs can significantly improve their specificity and correctness of feature characterization. Second, developing hybrid models combining the visual pattern recognition strengths of AI with structured clinical data can lead to more robust diagnostic systems. Third, AI techniques are required to provide trustworthiness so that clinicians can understand why the AI came to a particular conclusion—e.g., by highlighting the regions of the image that influenced its decision.

Lastly, the emergence of such technologies requires a paradigm shift in dental education. Futures curricula must include “AI literacy,” teaching students how to critically evaluate AI-generated reports, understand their inherent biases (e.g., to choose clearly defined, textbook lessons), and integrate this information into a comprehensive diagnostic process. Clinicians will have to be taught not to be passive recipients of AI output but to be active, critical interpreters with the ability to override faulty AI suggestions based on clinical judgment and experience.

## 5. Conclusions

This study demonstrates that, in the present evaluation setting, ChatGPT-5 exhibits a high-sensitivity, safety-oriented pattern for detecting potential periapical pathosis; however, its low specificity and limited agreement with expert assessments for lesion border characterization and selected morphological descriptors currently preclude its use as an independent diagnostic tool in endodontic practice. Model performance was strongly dependent on the presence of distinct radiographic characteristics, with preferential accuracy for overt and well-defined lesion patterns. The results further indicate that ChatGPT-5, in its current general-purpose configuration, cannot substitute for clinical expertise. Nevertheless, it may serve as a preliminary screening or triage-support tool, provided that all AI-generated findings are verified by qualified dental professionals. Future integration of multimodal large language models into clinical imaging workflows will require targeted domain-specific training on high-quality annotated dental datasets and rigorous external validation to improve morphological feature discrimination and reduce systematic over-diagnosis.

## Figures and Tables

**Table 1:** Descriptive summary of radiographic feature detection by ChatGPT and expert consensus (*n* = 256).

Parameter	Category	Expert Consensus n (%)	ChatGPT n (%)
Arch	Upper	58 (22.7%)	130 (50.8%)
	Lower	70 (27.3%)	94 (36.7%)
Position	Anterior	17 (6.6%)	30 (11.7%)
	Posterior	111 (43.4%)	194 (75.8%)
Tooth Involved	First Molar	49 (19.1%)	72 (28.1%)
	Second Molar	17 (6.6%)	16 (6.2%)
	Second Premolar	18 (7.0%)	26 (10.2%)
	Lateral Incisor	9 (3.5%)	28 (10.9%)
	Central Incisors	14 (5.5%)	14 (5.5%)
Borders	Ill-defined	103 (40.2%)	168 (65.6%)
	Well-defined, corticated	2 (0.8%)	24 (9.4%)
	Well-defined, non-corticated	23 (9.0%)	32 (12.5%)
Shape	Irregular	109 (42.6%)	187 (73.0%)
	Round	12 (4.7%)	26 (10.2%)
	Oval	7 (2.7%)	9 (3.5%)
Lamina Dura	Lost	254 (99.2%)	240 (93.8%)
	Intact	2 (0.8%)	16 (6.2%)
Root Resorption	Present	106 (41.4%)	68 (26.6%)
	Absent	150 (58.6%)	188 (73.4%)
Cortical Expansion	Present	42 (16.4%)	58 (22.7%)
	Absent	214 (83.6%)	198 (77.3%)
Tooth Displacement	Present	36 (14.1%)	56 (21.9%)
	Absent	220 (85.9%)	200 (78.1%)

**Table 2:** Diagnostic performance of ChatGPT compared with expert consensus for periapical lesion detection (n = 256).

Metric	Value
True Positives (TP)	112
True Negatives (TN)	16
False Positives (FP)	112
False Negatives (FN)	16
Accuracy	(50%)
Sensitivity (Recall)	(87.5%)
Specificity	(12.5%)
Precision (PPV)	(50%)
F1 Score	(63.6%)
Balanced Accuracy	(50%)

**Table 3:** Agreement between ChatGPT and expert consensus in the interpretation of periapical radiographic features (n = 256).

Parameter	Observed Agreement (%)	Cohen’s κ	Strength of Agreement
Detection (Normal/Abnormal)	50.0	0.000	Poor
Arch	92.9	0.857	Almost perfect
Position	90.2	0.589	Moderate
Tooth involved	56.2	0.464	Moderate
Borders	52.7	0.127	Slight
Shape	76.8	0.359	Fair
Lamina Dura	97.3	0.000	None
Root Resorption	45.5	0.014	Slight
Cortical Expansion	51.8	0.000	Poor
Tooth Displacement	95.5	−0.014	None

Cohen’s κκ was used to assess chance-corrected agreement between ChatGPT and expert consensus. Interpretation of κ follows the Landis & Koch (1977) [27] scale: <0 = Poor, 0.00–0.20 = Slight, 0.21–0.40 = Fair, 0.41–0.60 = Moderate, 0.61–0.80 = Substantial, 0.81–1.00 = Almost perfect.

**Table 4:** Comparative distribution between ChatGPT and expert consensus on abnormal radiographs.

Parameter	Category	Expert n (%)	ChatGPT n (%)	Test	χ^2^	*p*-Value
Arch	Lower	70 (54.7%)	54 (48.2%)	McNemar	1.12	0.289
	Upper	58 (45.3%)	58 (51.8%)			
Position	Anterior	17 (13.3%)	14 (12.5%)	McNemar	0.36	0.546
	Posterior	111 (86.7%)	98 (87.5%)			
Tooth Involved	First Molar ^a^	51 (39.8%)	46 (41.1%)	Bowker	12.7	0.078
	Second Molar ^a^	19 (14.8%)	8 (7.1%)			
	First Premolar ^a^	8 (6.2%)	2 (1.8%)			
	Second Premolar ^a^	18 (14.1%)	18 (16.1%)			
	Canine ^a^	1 (0.8%)	0 (0.0%)			
	Lateral Incisor ^a^	13 (10.2%)	16 (14.3%)			
	Central Incisor ^a^	14 (10.9%)	12 (10.7%)			
	Multiple Teeth ^a^	4 (3.1%)	4 (3.6%)			
	Premolar (unspecified) ^a^	0 (0.0%)	4 (3.6%)			
Borders	Ill-defined ^a^	103 (80.5%)	83 (74.1%)	Bowker	26.3	<0.001 *
	Well-defined, corticated ^b^	2 (1.6%)	9 (8.0%)			
	Well-defined, non-corticated ^ab^	23 (18.0%)	18 (16.1%)			
Shape	Irregular ^a^	109 (85.2%)	83 (74.1%)	Bowker	2.67	0.264
	Round ^b^	9 (7.0%)	9 (8.0%)			
	Oval ^ab^	10 (7.8%)	18 (16.1%)			
Lamina Dura	Lost	110 (85.9%)	110 (98.2%)	McNemar	2.0	0.156
	Intact	18 (14.1%)	2 (1.8%)			
Root Resorption	Present	58 (45.3%)	58 (51.8%)	McNemar	3.98	0.046 *
	Absent	70 (54.7%)	54 (48.2%)			
Cortical Expansion	Present	108 (84.4%)	108 (96.4%)	McNemar	1.22	0.270
	Absent	20 (15.6%)	4 (3.6%)			
Tooth Displacement	Present	111 (86.7%)	111 (99.1%)	McNemar	0.34	0.560
	Absent	17 (13.3%)	1 (0.9%)			

Superscripts (^a^, ^b^, ^ab^) indicate post hoc McNemar pairwise comparisons (*p* < 0.05, Bonferroni-adjusted). Categories sharing the same letter are not significantly different (*p* > 0.05). * significant overall Bowker/McNemar tests (*p* < 0.05). Percentages normalized to each rater’s own abnormal total (Expert n = 128; ChatGPT n = 112).

**Table 5:** Binary logistic regression analysis of ChatGPT radiographic, anatomical, and tooth-specific predictors of correct detection compared with expert consensus.

Predictor	Level (vs. Reference)	OR	95% CI	*p*-Value
Borders	Well-defined, corticated vs. Ill-defined	60.25	3.62–1002.64	<0.001 *
Borders	Well-defined, non-corticated vs. Ill-defined	3.46	1.49–8.04	0.004 *
Shape	Oval vs. Irregular	20.43	1.18–354.87	0.003 *
Shape	Round vs. Irregular	2.45	1.03–5.87	0.061
Lamina Dura	Intact vs. Lost	0.03	0.00–0.45	<0.001 *
Cortical Expansion	Present vs. Absent	2.10	1.02–4.31	0.046 *
Root Resorption	Yes vs. No	0.72	0.31–1.65	0.408
Tooth Displacement	Yes vs. No	0.89	0.45–1.78	0.725
Arch	Lower vs. Upper	1.61	0.96–2.68	0.092
Position	Anterior vs. Posterior	0.86	0.40–1.84	0.846
Tooth	Other vs. First Molar	32.55	1.92–551.74	<0.001 *

Exact odds ratios (OR) and 95% confidence intervals (CI) were derived using Fisher’s exact test with Haldane–Anscombe correction for sparse cells. An OR > 1 indicates greater odds that ChatGPT’s classification (normal vs. abnormal) matched the expert consensus; OR < 1 indicates reduced odds of agreement. * Statistically significant at *p* < 0.05.

## Data Availability

Research data supporting this publication is available from the corresponding author upon request.
